# No Severe Adverse Effects from Intravitreally Injected Putative Adipose Tissue-Derived Stem Cells

**DOI:** 10.1155/2019/6927829

**Published:** 2019-12-31

**Authors:** Carsten Faber, Steffen Heegaard, Jens Folke Kiilgaard

**Affiliations:** Department of Ophthalmology, University Hospital Copenhagen, Rigshospitalet, Denmark

## Abstract

This study reports findings from a 56-year-old patient, who had received bilateral intravitreal injection of putative adipose tissue-derived stem cells at a private clinic in India with the promise of treatment of NAION. During an observation period of 8, respectively, 18 months, the intravitreally injected cells remained silent in the vitreous bodies without either therapeutic effects or complications. The cells cleared with vitrectomy without evidence of integration in the optic nerve or retina. Contrary to recent reports on patients receiving intravitreal injections of similar putative stem cells with the aim of treating AMD, our patient suffered no devastating ocular consequences. *Summary*. Contrary to recent reports, this case demonstrated no devastating consequences of intravitreal injection of adipose tissue-derived stem cells during an observation period of up to 18 months. After vitrectomy, the cells cleared without evidence of either harm or integration.

## 1. Introduction

Stem cells have emerged as a promising therapeutic modality for multiple diseases [1–3]. Besides bone marrow and limbal transplantation, much needs to be learned before stem cell therapy can reach clinical practice. Despite this lack of knowledge about safety and efficacy, private clinics in several countries offer stem cell therapy promising treatment of multiple diseases.

Recent reports have described four cases of putative adipose tissue-derived “stem cells” injected intravitreally in patients (aged 72 to 88 years) with age-related macular degeneration in two different private clinics [4, 5]. While devastating consequences of the procedures, including retinal hemorrhage and detachment with proliferative vitreoretinopathy (PVR) in almost all injected eyes, were described in detail, the actual fate of the injected stem cells was not described.

## 2. Case

**Figure 1:**
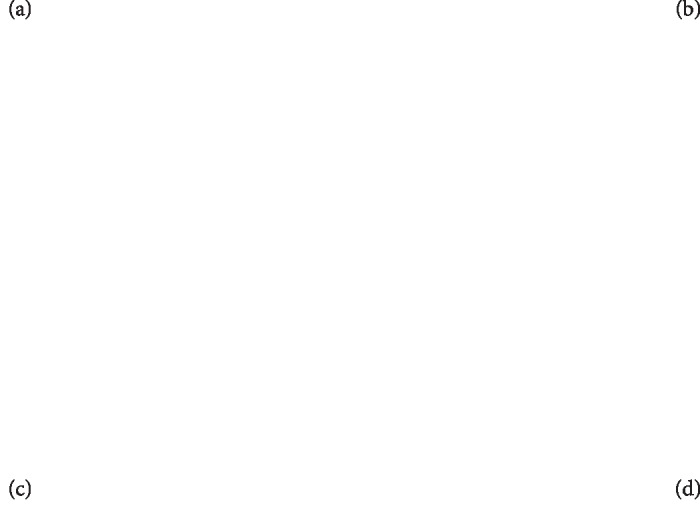
(a) Dense vitreal opacities are seen 2 months after intravitreal injection of adipose tissue-derived stem cells in the right eye. This condition remained unchanged during an 18-month observation period until vitrectomy was performed. (b) During vitrectomy, the dense opacities were seen localized to the vitreous body; no retinal integration or reaction was evident. (c) Two months after vitrectomy, the eye remained silent with unchanged epiretinal remnants from the adherent hyaloid. The left eye had similar findings before, during, and after vitrectomy.

A 56-year-old male with vision loss from sequential nonarteritic anterior ischemic optic neuropathy (NAION) with a complete visual field defect and a visual acuity of 1/50 Snellen in each eye (Figure 1(a)) was seen acutely due to a further, painless decrease in visual acuity in both eyes. A visual acuity of light perception was noted. On examination, whitish, dense vitreal opacities partly obscuring views of deeply atrophic optic nerves and otherwise unremarkable retinas were seen. The conjunctivae were white and no anterior uveitis was seen.

The patient revealed that two months earlier he received intravitreal injections in both eyes of putative stem cells prepared from his abdominal subcutaneous adipose tissue. The full procedure from harvesting of adipose tissue to intravitreal injection had a total duration of about two hours and was carried out at a private clinic in New Delhi, India. Further details about the procedure were not known.

The patient was observed and demonstrated a stable condition for several months (Figure 1(a)). Eight months after stem cell injection, we performed vitrectomy in the left eye and—after a further 10 months—also in the right eye. The dense opacities were purely localized to the vitreous bodies and cleared completely with vitrectomy (Figure 1(b) and video, supplementary material ([Supplementary-material supplementary-material-1])). The hyaloids were very adherent to the retinae which probably was the cause of the intraoperative complications: in the left eye a localized retinal detachment was induced and in the right a retinal tear was induced, both were treated accordingly. Specimens from both eyes were examined microscopically and included few vimentin-positive cells compatible with cells of mesenchymal origin. We did not identify cells differentiated into a retinal or neuronal phenotype. Following the immediate postoperative period, no intraocular reaction was observed in either eye. Visual acuity remained poor. Epiretinal remnants from the adherent hyaloid were noted in the right eye, and these remained unchanged for several months (Figure 1(c)).

## 3. Discussion

This case demonstrates that cell suspensions of putative stem cells may be injected intravitreally without severe adverse effects. Whether this was due to entrapment in the vitreous body or simply lack of function of putative stem cell population is not known. The posterior hyaloid was very adherent in both eyes, which may be an effect from or a response to the intravitreally placed cell population. We did, however, not note any signs of cellular integration in the retina or the optic nerve and certainly no therapeutic effect was noted.

The cell suspensions remained intravitreally for 8-18 months. Besides unchanged vitreal clouding, no serious adverse effects were noted in the observation period. In agreement with the immune privilege of the eye including the vitreous, this demonstrates that heterogeneously transplanted cells can remain silent without inducing an inflammatory response in the vitreous. This is unlike previous reports where intravitreal injections of similar adipose tissue-derived cell populations resulted in early and serious complications in all treated eyes. This included high intraocular pressure, anterior uveitis, lens subluxation, vitreal and intraretinal hemorrhage, and retinal detachment with aggressive PVR [4, 5]. This difference may relate to different methods of harvesting and isolating adipose tissue-derived stem cells resulting in debris or toxicity inducing these reactions rather than a reaction to the cell isolate per se. We speculate that another explanation for the observed clinical difference could be the absence of posterior vitreous detachment. In the present case, cell suspensions were probably injected into the persistent vitreous bodies. In the previous cases, all patients were older than 70 years with end-stage AMD, and the cell suspensions were probably injected in close contact with the retina due to posterior vitreous detachment.

The stem cell technique holds great promise for treatment and cure of a wide range of diseases; however, more research is needed. Currently, some private clinics, with a promise of cure, are exploiting patients with untreatable diseases. This case demonstrates that patients should be discouraged from such unethical and costly treatments.

## Supplementary Materials

Supplementary Materials Video of the vitrectomy of the right eye.
